# Multicomponent Exercise Intervention for Preventing Falls and Improving Physical Functioning in Older Nursing Home Residents: A Single-Blinded Pilot Randomised Controlled Trial

**DOI:** 10.3390/jcm13061577

**Published:** 2024-03-10

**Authors:** Munseef Sadaqa, Wesam A. Debes, Zsanett Németh, Zsófia Bera-Baka, Marianna Vachtler-Szepesi, Loretta Nácziné Földes, Viktória Prémusz, Márta Hock

**Affiliations:** 1 Doctoral School of Health Sciences, Faculty of Health Sciences, University of Pécs, Vorosmarty Street 4, 7621 Pécs, Hungary; munseef.sadaqa@etk.pte.hu (M.S.); flk0r5@pte.hu (W.A.D.); zsani0008@gmail.com (Z.N.); hock.marta@etk.pte.hu (M.H.); 2 Aranykor Nursing Home, Aranykor Road 38, 6035 Ballószög, Hungary; bakazsofi@gmail.com (Z.B.-B.); szepesi.marianna3@gmail.com (M.V.-S.); foldeslotty9506@gmail.com (L.N.F.); 3 Physical Activity Research Group, Szentágothai Research Centre, Ifjúság Road 20, 7624 Pécs, Hungary; 4 Institute of Physiotherapy and Sport Science, Faculty of Health Sciences, University of Pécs, Vörösmarty Street 3, 7621 Pécs, Hungary

**Keywords:** falls, physical function, older people, frailty, nursing home, long-term care facility, strength exercise, balance exercise, multicomponent exercise, randomised controlled trial

## Abstract

**Background:** Older nursing home residents are at a greater risk of falling due to frailty. Exercise is effective at hampering frailty and related adverse events, including falls. Objectives: Our purpose was to evaluate the effect of a 12-week moderate-intensity multicomponent exercise programme on the number of falls and physical functioning among older nursing home residents. Also, we examined the association between the number of falls and demographics as well as physical and cognitive baseline data. **Methods:** The study protocol was registered on clinicaltrials.gov with the following identifier: NCT05835297. Older adults aged 65 years and over were recruited from a nursing home, and eligible and consenting residents were randomly allocated to two parallel groups: the intervention group, which performed a multicomponent exercise programme composed of strength, balance, and aerobic training (*n* = 12), and the control group, which received usual care (*n* = 12). Outcomes included falls, and measures of strength, balance, and mobility. **Results:** We had high adherence to exercise sessions, and no adverse events were recorded. We observed a non-significant reduction in falls (*p* = 0.34) and a significant improvement in Short Physical Performance Battery (*p* = 0.003) after the exercise programme. Falls were associated with being female and having diminished physical or cognitive function. **Conclusions:** Multicomponent exercise programmes should be implemented regularly in nursing homes for their effectiveness. Future studies with bigger samples, including participants with worse physical and cognitive impairments, as well as follow-up periods are required.

## 1. Introduction

The older population is at an increased risk of developing frailty; a geriatric syndrome that deepens the emergence of several negative health-related events, including falls. Frailty is also associated with comorbidity and disability [1,2]. Falls and their related sequelae are considered to be global health concerns affecting the population of older adults. As much as 35% of community-dwelling individuals aged 65 or older fall at least once a year, and this figure increases to 50% of individuals over the age of 80 [3,4]. Among long-term care facility (LTCF) residents, the rate increases up to 50%; 12–40% of them experience recurrent falls [5]. The aetiology of falls is believed to be multifactorial; they occur as a result of complex interactions between both intrinsic and extrinsic risk factors [3,6,7]. Extrinsic factors can be environmental (e.g., slippery floors and stairs, and insufficient lighting), or socioeconomic (e.g., low income and education levels, and lack of social interactions) [3]. Amongst the intrinsic risk factors, many are related to physical capacity, including loss of muscle mass, strength, and function (sarcopenia), impaired balance, and reduced mobility [6,8]. These risk factors are also embedded within the concept of frailty, which is an aggravating factor for falls and a leading cause of disability [1,9]. In addition to these factors among older adults in general, LTCF residents are older than those living in communities; therefore, they are frailer, having higher comorbidity and cognitive deficits; polypharmacy is more prevalent among them, as well as living with higher dependency in performing Activities of Daily Living (ADLs); these are further potential factors that heighten their vulnerability to falls and fall-related injuries [10,11,12]. 

Moreover, older individuals in LTCFs typically lead a sedentary lifestyle and scarcely exercise (mostly once per week or even less); that plausibly augments the decline in their physical functioning [13]. Furthermore, in relation to LTCFs, falls can be described as being of a bidirectional nature since they might be a cause of or a result of admission into LTCFs [14,15]. According to the World Health Organization report on falls prevention, death is not the most common consequence of falls; 40% of all accidental deaths among older people were ascribed to falls [3]; however, 5–10% of falls lead to serious injuries such as head injuries or fractures [16]. The impacts of falls are not restricted to harmful physical ones but also can be psychological ones; for instance, fear of falling, depression, anxiety, and cognitive deficits [17,18,19]. Falls are also a major public health problem; they create a substantial financial burden for the healthcare system because of the considerable related healthcare expenditures [20]. Studies have shown that exercises are effective in the prevention of falls, attenuation of their related risk factors, as well as improving the quality of life of older adults [21,22,23]. Explicitly, the impact of multicomponent exercise interventions on improving or maintaining physical functions, for example, muscle strength, mobility, and balance, as well as reducing falls for community-dwelling older people, is continuously being investigated and updated in the literature, and findings are substantially consistent [24,25,26,27]; however, the studies on LTCF residents are relatively contradictory and inconclusive [7,28,29,30,31,32,33,34,35,36,37,38,39,40,41,42,43,44,45]. 

Multicomponent exercise programmes composed of balance, muscle strengthening, and aerobic training and performed at moderate intensity are recommended by the International Association of Gerontology and Geriatrics-Global Aging Research Network (IAGG-GARN) and the IAGG-European Region Clinical Section taskforce; they have been shown to be effective in improving ADL performance in older adults living in LTCFs [46].

However, a limited number of Randomised Controlled Trials (RCTs) have focused on the evaluation of multicomponent exercise interventions performed for short durations at a moderate intensity in LTCF settings; these studies revealed that similar exercise programmes are effective in reducing falls and improving the parameters of physical functions such as muscle strength, gait ability, and balance performance [28,42,44]. 

Moreover, no RCT has assessed the effect of a multicomponent exercise programme on falls and physical functioning parameters as proposed by IAGG-GARN and the IAGG-European Region Clinical Section. 

To fill these gaps in the literature, in the present pilot study, we aimed to evaluate the effect of a 12-week moderate-intensity multicomponent exercise programme on the number of falls and physical functioning among LTCF residents aged 65 years and over. Additionally, we investigated the association between the number of falls and various baseline data.

## 2. Materials and Methods

### 2.1. Study Design

This 12-week single-blind, parallel, pilot RCT was approved by the Institutional Review Board/Regional Research Ethics Committee of the University of Pécs, the Medical Research Council Hungary, and the National Public Health Centre (Record number 9283-PTE 2022), and registered on clinicaltrials.gov with the identifier NCT05835297. The protocol conformed to the Standard Protocol Items: Recommendations for Interventional Trials (SPIRIT) statement [47]. The trial was designed under the Declaration of Helsinki, and reported according to the CONsolidated Standards Of Reporting Trials (CONSORT) statement [48]. 

### 2.2. Recruitment and Eligibility

**Figure 1:**
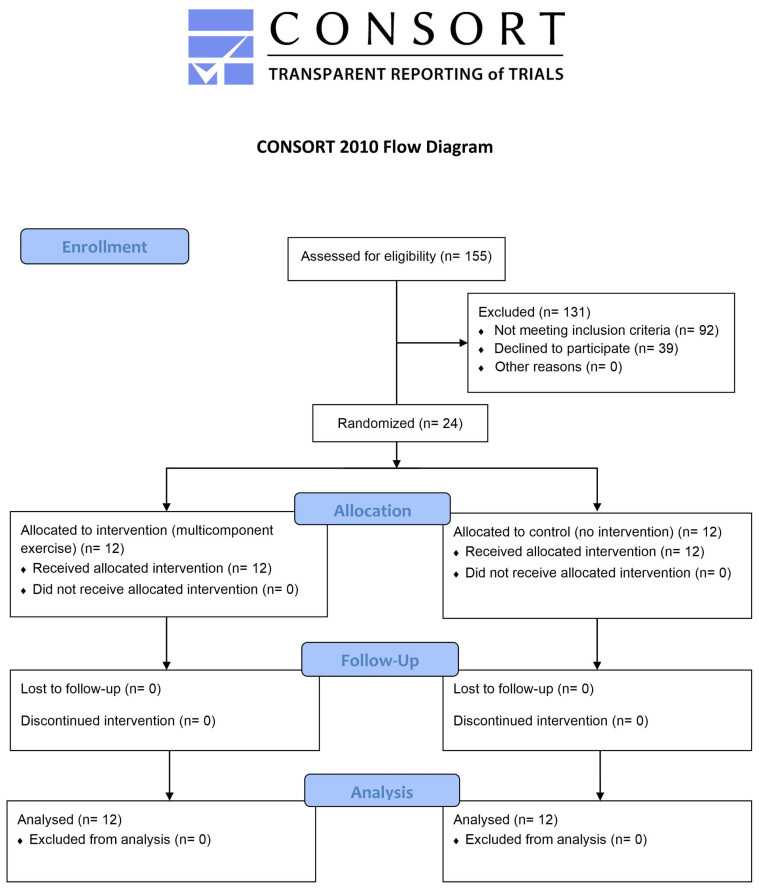
CONSORT study flow diagram.

Individuals aged 65 years and over were recruited from a nursing home in Hungary. Following the eligibility assessment, participants provided written informed consent before they were randomly allocated to either the intervention group (IG) or the control group (CG); then, baseline assessments were performed to avoid a long interval between participant assessment and the beginning of the interventions. Figure 1 depicts the study flow diagram.

Eligible participants for the trial were required to comply with the following criteria: aged 65 years or older, living in the nursing home; physically mobile (capable of ambulating/rising from a chair with or without assistance); not being under simultaneous specific physical activity/exercise investigations in other experimental studies or under other specific exercise rehabilitation programme; ability to follow verbal instructions. Exclusion criteria included being physically unable or medically unfit to participate in physical exercise after medical consultation and a Mini-Mental State Examination (MMSE) score < 18.

### 2.3. Randomisation

Participants were randomised through stratified randomisation based on age, sex, and baseline values of Short Physical Performance Battery (SPPB) to either IG or CG, using a computer-generated method [http://www.randomizer.org, accessed on 27 March 2023] by an independent researcher who was blind to both intervention and assessments to ensure allocation concealment.

### 2.4. Intervention Group Activities

The group-based multicomponent exercise intervention at moderate intensity consisted of strength, balance, and aerobic exercises for older adults living in LTCFs; the exercise programme was designed based on the recommendations of the IAGG-GARN and the IAGG European Region Clinical Section on exercises for older adults living in LTCFs [46]. The programme followed 12 weeks of supervised sessions at the nursing home, conducted twice a week, on non-consecutive days for 45–60 min per session by physiotherapists working at the facility, as proposed by the IAGG-GARN.

The exercise session comprised five min of warm-up (e.g., range of motion exercises of upper and lower extremities, followed by light walking), 10 min progressive static and dynamic balance exercises (e.g., semi-tandem, tandem, single-leg stand, reaching forward, walking in a line, tandem walking in a line, walking with changing directions, and walking forward, backward, and sideways along straight line), 15–20 min strength exercises performed through weight-bearing exercises and using free weights (e.g., one or two sets of 13–15 repetitions maximum of chair rises, knee extension and flexion, and heel raises); however, during the first week, low-intensity exercises with repetitions maximum up to 20 were performed with progression in intensity (i.e., increase speed of movement, change to a lower chair, and hold weight in hands), 15–20 min aerobic exercises (e.g., five 3 min bouts of walking between two strengthening exercises and/or between two balance exercises), and five min of cool down exercises of light walking and stretching exercises. Exercise intensity is intended to be moderate. When an individual improves the execution of an exercise, a progression was proposed by increasing exercise difficulty, duration of exercise, the number of repetitions to be performed, or exercise load [46,49,50,51,52,53,54]. 

### 2.5. Control Group Activities 

Participants allocated to the CG received usual care and participated in routine low-intensity activities usually offered to the residents at the nursing home. 

### 2.6. Primary and Secondary Outcome Measures

Trained assessors blinded to group allocation conducted outcome assessments at baseline and at 12 weeks. The primary outcome measure in this study was the number of falls. Falls were defined as “inadvertently coming to rest on the ground, floor or other lower level, excluding intentional change in position to rest in furniture, wall or other objects” [3]. We collected participants’ falls during the three months prior to enrolment (baseline falls) with sociodemographic data, and for falls during the intervention period, the staff involved used a daily diary to record any fall event in an incident report that included the name of the faller, date, and time of fall [55]. 

Secondary outcomes included lower extremity functionality as evaluated using Short Physical Performance Battery (SPPB) that consists of standing balance, gait ability, and leg strength tests. Participants were required to stand stably in an upright position under three conditions: legs closed/feet together, semi-tandem stand, tandem stand for 10 s. Then, comfortable gait speed was assessed by measuring the time to walk a 4 m track, starting from a standing position, and stopping when the first foot was past the 4 m line. Finally, a sit-to-stand transfer was completed five times as fast as possible. 

Each test is scored between 0 and 4, and the overall SPPB overall score was created by summing the scores of the three tests and ranging from 0 (low mobility/functionality) to 12 (full mobility/functionality). Low scores of the tests were strongly associated with disability and increased risk of death among older adults [56]. 

A 6 min Walking Test (6MWT) evaluated physical endurance and mobility; the test yielded reliable and valid measures of physical endurance in older adults [57]. The participants were instructed to walk the longest distance possible within 6 min. Participants were allowed to stop and rest in a chair placed along the walking course, but the time did not stop. Total distance walked was recorded in meters. 

Timed Up and Go (TUG) test measured functional mobility, and is a valid and reliable tool for assessing mobility in older people [58]. The score is the time in seconds taken to stand up from a chair (seat height 47 cm), walk 3 m, turn, return to the chair, and sit down. Participants walked with their usual walking aids, if needed, at a comfortable speed. 

Single Leg Stance (SLS) test evaluated the static balance capability, and it showed significance in predicting falls among older adults [59]. The participants were asked to stand on one leg with eyes open on a firm surface for as long as possible; the time in seconds was recorded. 

Functional Reach Test (FRT) evaluated dynamic balance, a reliable, precise, and reasonable measure for detecting balance impairment in older people [60]. With a levelled yardstick placed on the wall, participants were instructed to stand next to the wall and position the arm that is closer to the wall at 90 degrees of shoulder flexion with a closed fist. The assessor records the starting position on the yardstick, then the subjects were asked to reach as far as they can forward without taking a step and the end position was recorded on the yardstick again. Scores were determined by assessing the difference between the start and end position in cm. Three trials were performed and the average of the trials was recorded [61]. 

In addition to the previously mentioned measures, we collected the following participant characteristics in order to describe the studied sample: age, sex, Body Mass Index (BMI), which was calculated after collecting the weight and height of participants (BMI = weight (kg)/height (m^2^)), and cognitive function using the MMSE score, which was retrieved from the participants’ records as it is consistently updated for the residents at the nursing home. Impaired cognition measured by MMSE was associated with falling among older adults in nursing homes [62].

### 2.7. Adherence and Adverse Events

Adherence to exercise was reported by study staff and was determined by the number of sessions attended. To promote retention, we clearly communicated to participants the aim of the study, ensured that the training area was comfortable and safe for them, and reminded them of their scheduled training session; the study staff also received their feedback and inquiries continuously. 

Adverse events, including falls, cardiac events, and stroke, were documented by study staff throughout the 12-week intervention period. Safety during sessions was ensured by closely supervised group exercise sessions of a maximum of six participants, which were always conducted by two physiotherapists. Additionally, chairs were available either next to or in front of the participants for resting on or grasping while performing the exercises. 

Participation was completely voluntary; participants could withdraw from the study at any time without any reason and cost, and this would not have affected care, services, or benefits to which they were entitled.

### 2.8. Sample Size Estimation

The sample size required to power a future RCT was calculated after considering the following: a possible loss to follow-up of 20% and a mixed design analysis of variance (ANOVA) to detect between-group differences, with an estimated effect size of 0.20, and a significance level adopted as 0.05, statistical power of 0.80, and correlation of 0.80 between measures (pre- and post-test); therefore, we will aim to recruit a sample of 80 participants, with 40 participants per group [63]. G*Power software (version 3.1.9.7; Heinrich-Heine-Universität Düsseldorf, Düsseldorf, Germany) was used for sample size calculation.

### 2.9. Statistical Analyses

Descriptive statistics were presented as means and Standard Deviations (SDs) for participants’ characteristic variables, as well as for primary and secondary outcomes. 

The Shapiro–Wilk test was used to evaluate the normal distribution of the data. Demographics and baseline data statistical comparisons between groups were performed using independent sample *t*-test and Chi-squared test for normally distributed data, while Mann–Whitney U-test was run for not normally distributed ones. A two-way repeated measures analysis of variance was conducted to examine between-group differences in mean change from baseline to 12 weeks. Within groups, differences were analysed using paired sample *t*-test for normally distributed data and Wilcoxon test for not normally distributed data. Poisson regression was implemented for assessing the effect of baseline data on number of falls during the study period and the three-month period prior to the beginning of the study. The significance level for all tests was set at *p* < 0.05. Statistical analyses were performed using IBM SPSS Statistics for Windows, version 27.0 (IBM SPSS, Armonk, NY, USA: IBM Corp.).

## 3. Results

No significant differences between the groups were detected in the participant baseline demographics and physical functioning measures (Table 1 and Table 2).

### 3.1. Adherence and Adverse Events

The attendance rate for the exercise sessions was 100%. No adverse events (cardiac events, stroke, or falls) occurred during the exercise sessions. 

### 3.2. Falls

For the number of falls during the intervention, the CG (*n* = 12) had a larger mean rank (13.4) (a higher number of falls) than the IG (*n* = 12) with a mean rank of (11.6); however, a Mann–Whitney U test indicated that this difference was not statistically significant: U = 61.5, *p* = 0.35. 

**Figure 2:**
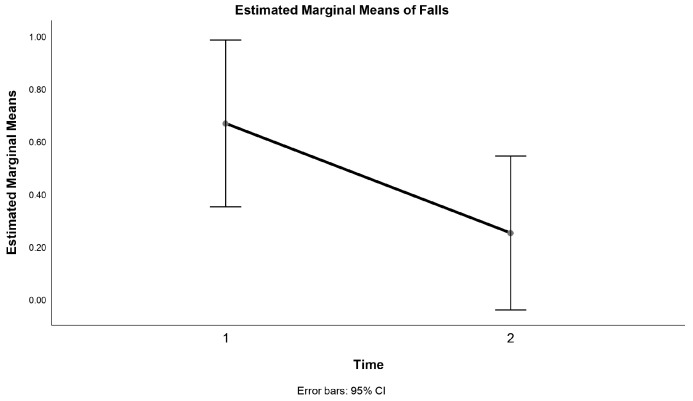
Estimated marginal means of falls.

Moreover, the IG as well as the CG had a lower number of falls during the intervention compared to three months before the intervention, yet in the IG, the Wilcoxon test revealed that the difference was not statistically significant: T = 6.0, z = −0.9, *p* = 0.34; however, the CG showed a significant decrease *p* < 0.05 (Table 2). In contrast, the Wilcoxon test indicated that a 12-week exercise intervention elicited a statistically significant difference in the number of falls between the 12 weeks preceding the intervention and during the intervention, irrespective of the group: T = 16.5, z = −2.3, *p* = 0.02 (Figure 2).

A Poisson regression was run to determine which of the baseline variables, including sex and physical and cognitive functions, have a statistically significant effect on the number of falls that a participant experiences throughout the study period, as well as the last three months before commencing the study.

The test showed the number of falls during the three months preceding the study was significantly predicted by sex (*p* = 0.012, 92.2% lower in males), pre-SPPB (13.8% decrease in falls for 1 unit increase in the SPPB score, *p* = 0.035), pre-6MWT (0.3% decrease in falls for 1 unit increase in pre-6MWT, *p* = 0.038), pre-SLS test-dominant (26.8% decrease in falls for 1 unit increase in pre-SLS test-dominant, *p* = 0.003), pre-SLS test-non-dominant (15.9% decrease in falls with 1 unit increase in pre-SLS test-non-dominant, *p* = 0.029), MMSE (10.9% decrease in falls with 1 unit increase in MMSE, *p* = 0.018), and marginally significant for pre-FRT (4.6% decrease in falls with 1 unit increase in pre-FRT, *p* = 0.050).

For the number of falls during the study period, the test revealed that it is significantly predicted by the pre-SPPB (for 1 unit increase in SPPB score, falls decrease 35.3%, *p* = 0.019), pre-6MWT (for 1 unit increase in 6MWT score, falls decrease 1.3%, *p* = 0.016), pre-TUG test (for 1 unit increase in TUG test score, falls increase 13.6%, *p* = 0.016), and marginally significant for the MMSE (for 1 unit increase in the MMSE score, falls decrease by 20.3%, *p* = 0.056).

### 3.3. Physical Functioning

**Figure 3:**
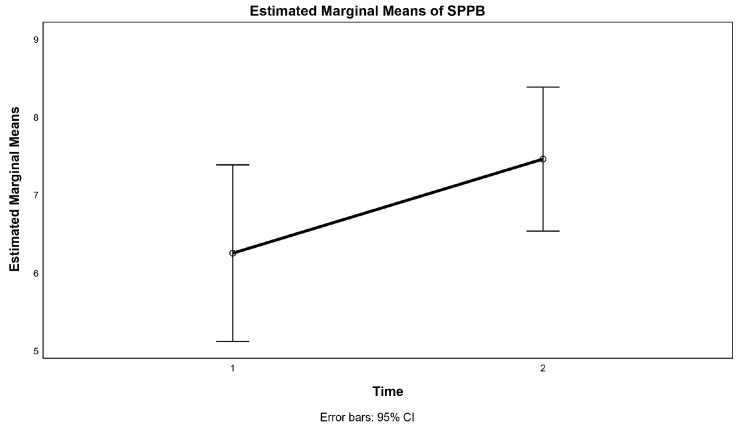
Estimated marginal means of SPPB.

After 12 weeks of the exercise intervention, the time-by-group interaction in the mixed design ANOVA was not statistically significant in any physical functioning measure (Table 2); however, there was a significant difference between the two time points of the SPPB score, irrespective of the group, F (1.22) = 8.67, *p* = 0.007, η2 = 0.283 (Figure 3).

When the differences were analysed within groups by a paired *t*-test, the IG had a significantly improved SPPB score, t (11) = 3.78, *p* = 0.003, whereas the remaining outcome measures, except for SLS test-dominant, have been improved compared to the baseline assessment, yet not significantly (Table 2). In CG, there was no significant change for any outcome measure.

## 4. Discussion

The present study investigated the effect of a 12-week moderate-intensity multicomponent exercise programme consisting of strengthening, balance, and aerobic training on falls and physical functioning among older LTCF residents aged 65 years or over. Additionally, we investigated the association between the number of falls and baseline variables, including sex and physical and cognitive functions. The exercise programme enhanced physical functioning parameters as well as falls reduction among the IG participants. Falls in our study were associated with being female, as well as having diminished physical or cognitive function. 

Despite the number of falls being lower in the IG compared to the CG during the intervention, the difference between the two groups was not significant. Regarding the differences in the number of falls between pre-intervention and during the intervention, even though both groups had lower numbers of falls, the reduction was only significant in the CG. Nevertheless, there was a significant difference in the number of falls in this outcome for all the participants, regardless of the group.

The lack of a significant effect of the exercise programme on falls reduction in the present study complies with the results of other studies, even though they had individualised exercise programmes and relatively bigger sample sizes and doses than ours [28,34,36]. This was attributed to the small sample size, the short duration of the programme [34], and that falls could have occurred before the attainment of maximum exercise effects [36].

The significant reduction in falls for the CG might have occurred for two reasons. First, the Hawthorne effect, in which residents in this group modified their behaviours and became more vigilant since they knew they were being observed and were aware of group allocation [64]. Second is the risk of contamination between the groups; either residents in the IG communicated the exercises to the residents in the CG, or the latter might have accidentally observed a number of sessions for the IG, then became aware of the exercises and performed them [65,66].

Nevertheless, our study outlined that shorter interventions have effects on falls, despite being not significant. Likewise, short-duration interventions showed effectiveness in reducing falls, which are credited to the improvement in the strength of lower limbs via the strengthening and balance components in the programmes [28,33]. However, Wang and Tian, in their recent systematic review and meta-analysis, concluded that simple long-term exercise interventions are more effective in falls prevention in LTCF, as a result of improvements in muscle strength and balance [31]. 

Studies have reported the effectiveness of individualised multicomponent exercises at a moderate intensity in preventing falls in LTCF residents [30,38], and gains were lost after the exercises were halted [30,39]. Still, not only individualised exercises effectively prevented falls, but also non-individualised group exercises [7,31]. 

Since falls and their consequent injuries are more prevalent among Long-Term Nursing Home (LTNH) residents, the importance of exercise interventions to prevent increased falls in this setting has been highlighted in different studies as well [7,67,68]. 

Based on the baseline data in our study, we found that the number of falls was significantly associated with sex (being female), functional measures of strength, balance, and mobility, as well as cognitive measures.

Consistent with a previous study, this study found that being female is associated with an increased likelihood of falling [69]; a possible explanation lies in the differences in the intrinsic factors between males and females [70]. On the contrary, others found that males are more likely to fall compared to females [62,71,72]; as males are losing more testosterone and muscle mass, as well as facing a decline in muscle strength during the course of ageing at a more rapid pace compared to females, this increases their vulnerability to sarcopenia and, consequently, to falls [73,74,75]. 

Furthermore, our analysis revealed that impaired functionality is a predictor of falling among LTCF residents. Other studies have reported that falls were significantly higher with increased frailty, as well as impaired balance and mobility [62,71,72]. In contrast, other studies have revealed that higher frailty levels and a decline in mobility were protective factors for falls and fall-related hospitalisations [71,76]. Moreover, while in some studies, dependency in ADL performance accounted for a risk factor for falls [16,72], others found it to be a protective factor [77]. These might be explained by the fact that older residents with increased frailty and diminished mobility ambulate less; therefore, they have lesser exposure to physically challenging situations and, consequently, have a reduced likelihood of falling [76,77].

The present study confirmed the previously established association between lower cognitive function and increased likelihood of falling [62,71,72,78]. On the contrary, Castaldo et al. observed that good cognitive function and mild to moderate cognitive impairment are associated with more frequent falls among LTCF residents; they argued that individuals with severe cognitive impairment ambulate less and consequently have reduced encounters with fall-leading situations. Additionally, they might observe greater vigilance from the staff compared to with residents with better functionality [77]. 

In our study, the 12 weeks of a moderately intensive multicomponent exercise programme were adequate to show improvements in physical functioning outcome measures for the IG. At the same time, for the CG, there was a decline in most of them. 

We observed a significant difference in the SPPB score at the end of the 12-week intervention compared to the pre-intervention SPPB score in the IG. This significant improvement is in line with the results of previous studies [30,40,41,42,43]. SPPB is one of the tools that are used to assess frailty in older adults [30,79,80], which is highly prevalent among LTNH residents [81,82], and it is related to weakness and reduced physical activity, eventually resulting in impaired physical functionality and an increased risk of falls [2,8,83].

Although, in our study, the only improvement in the SPPB was significant among the physical functioning outcome measures, others have reported significant improvements in a wide variety of measures after multicomponent exercise interventions, including a 30 s chair-stand test, the Berg balance scale, TUG test, gait speed, standing speed, and Barthel Index, as well as effectiveness in reducing frailty and its related adverse outcomes such as falls, disability, and mortality [30,33,42,43,44]. Still, effectiveness has been shown after short-duration programmes [42,43,44] and maintained after a long detraining period [43].

A recent systematic review and meta-analysis concluded a robust benefit of exercise interventions exists on physical functionality in LTCF residents with different functional and cognitive abilities. Moreover, the study indicated that the optimum gains were achieved by exercising for about three hours a week [45].

A major strength of this investigation resides in its distinctive position as one of the scarce RCTs delving into the impact of a brief yet moderate-intensity multicomponent exercise regimen on the prevention of falls and enhancement of physical functioning within an LTCF setting. A second major strength is that, to our knowledge, it is the first RCT to examine the exercise recommendations of IAGG-GARN and the IAGG-European Region Clinical Section for falls prevention and improvement in physical functionality in the LTCF setting as well. Furthermore, the high adherence to the exercise programme and the total absence of adverse events are also among the strengths of the present study. Finally, the exercise programme followed is relevant and adaptable for application in facilities with various preparedness.

Nevertheless, our study presents the following limitations. First, the sample size was small and the participants were recruited from a single facility; therefore, the results cannot be extrapolated to all residents of LTCFs, taking into consideration the heterogeneity in their physical and cognitive functions. Second, no follow-up test was conducted to examine the long-term detraining (inactivity) effect. Third, recall bias might have occurred when the participants reported their falls during the three months before the study. Finally, there was a possible contamination bias between the groups in the LTCF setting. 

## 5. Conclusions

In summary, the exercise intervention implemented in the present pilot RCT was both feasible and safe. The high adherence to the exercise sessions implies that the exercise programme was well-tolerated by the IG participants and enhanced their physical functioning parameters as well as falls reduction. Falls in our study were associated with being female, as well as having diminished physical or cognitive function. In the everyday context, considering the beneficial effect of exercise interventions composed primarily of strengthening and balance exercises, with aerobic and gait training on older adults in LTCFs, they should be emphasised in the routine practice in this setting. However, future studies with bigger sample sizes are required to establish the effectiveness of the exercise programmes for LTCF older residents, as well as for residents with greater physical or cognitive decline. A follow-up assessment to evaluate the impact of the detraining period on falls and physical function parameters is also suggested.

## Figures and Tables

**Table 1:** Characteristics of study participants at baseline (mean ± SD).

Variable	Intervention Group (*n* = 12)	Control Group (*n* = 12)	*p*-Value
Age (years)	78.3 ± 7.0	78.5 ± 7.4	0.93
Sex n (%)			
Male	3 (25)	4 (33.3)	1.00
Female	9 (75)	8 (66.7)	
BMI (kg/m^2^)	24.1 ± 5.3	25.7 ± 3.8	0.41
Falls in previous 3 months	0.4 ± 0.6	0.9 ± 0.8	0.14
MMSE score	26.1 ± 3.1	25.3 ± 3.9	0.68

Abbreviations: BMI = Body Mass Index; MMSE = Mini-Mental State Examination; SD = Standard Deviation.

**Table 2:** Falls and physical functioning outcomes (mean ± SD) following exercise intervention ^††^.

Variable	Intervention Group (*n* = 12)		Control Group (*n* = 12)		Time × Group Effect
	Pre	Post	Pre	Post	
Report of falls	0.4 ± 0.6	0.3 ± 0.9	0.9 ± 0.8	0.3 ± 0.5 ^§^	0.23
SPPB score	5.9 ± 2.7	7.7 ± 1.9 **	6.6 ± 2.6	7.3 ± 2.4	0.20
6MWT (m)	223.1 ± 137.9	235.8 ± 133.8	251.3 ± 92.2	200.9 ± 123.3	0.08
TUG (s)	18.6 ± 9.2	16.9 ± 9.5	15.8 ± 4.1	18.7 ± 8.1	0.97
SLS dominant (s)	2.5 ± 1.7	2.0 ± 1.3	3.5 ± 3.1	2.4 ± 1.4	0.16
SLS non-dominant (s)	3.4 ± 4.9	2.8 ± 2.9	3.3 ± 3.6	1.1 ± 0.9	0.26
FRT (cm)	34.5 ± 4.3	36.5 ± 8.1	33.9 ± 11.8	32.4 ± 6.0	0.43

Abbreviations: 6MWT = 6-min Walking Test; BMI = Body Mass Index; FRT = Functional Reach Test; SD = Standard Deviation; SLS = Single Leg Stance; SPPB = Short Physical Performance Battery; TUG = Timed Up and Go. ^††^ No significant differences between groups at baseline, *p* > 0.05 by independent *t*-test and Mann–Whitney U test; ^§^ *p* < 0.05, significant difference from baseline by Wilcoxon test; ** *p* < 0.01, significant difference from baseline by paired *t*-test.

## Data Availability

The data presented in this study are available on request from the corresponding author. The data are not publicly available due to privacy restrictions.
